# CT Scanning Imaging Method Based on a Spherical Trajectory

**DOI:** 10.1371/journal.pone.0149779

**Published:** 2016-03-02

**Authors:** Ping Chen, Yan Han, Zhiguo Gui

**Affiliations:** 1 National Key Laboratory for Electronic Measurement Technology, North University of China, Taiyuan 030051, China; 2 Key Laboratory of Instrumentation Science & Dynamic Measurement, North University of China, Taiyuan 030051, China; Shanxi University, CHINA

## Abstract

In industrial computed tomography (CT), the mismatch between the X-ray energy and the effective thickness makes it difficult to ensure the integrity of projection data using the traditional scanning model, because of the limitations of the object’s complex structure. So, we have developed a CT imaging method that is based on a spherical trajectory. Considering an unrestrained trajectory for iterative reconstruction, an iterative algorithm can be used to realise the CT reconstruction of a spherical trajectory for complete projection data only. Also, an inclined circle trajectory is used as an example of a spherical trajectory to illustrate the accuracy and feasibility of this new scanning method. The simulation results indicate that the new method produces superior results for a larger cone-beam angle, a limited angle and tabular objects compared with traditional circle trajectory scanning.

## Introduction

X-ray CT (computed tomography) imaging is widely used in industrial NDT (non-destructive testing) and clinical diagnosis [1][2]. However, for some complicated and irregular objects, there may be a mismatch between the thickness of the object and the ray energy along the direction of ray attenuation. This mismatch results in projection loss because of the physical limitation posed by the dynamic range of the CT imaging system.

**Fig 1:**
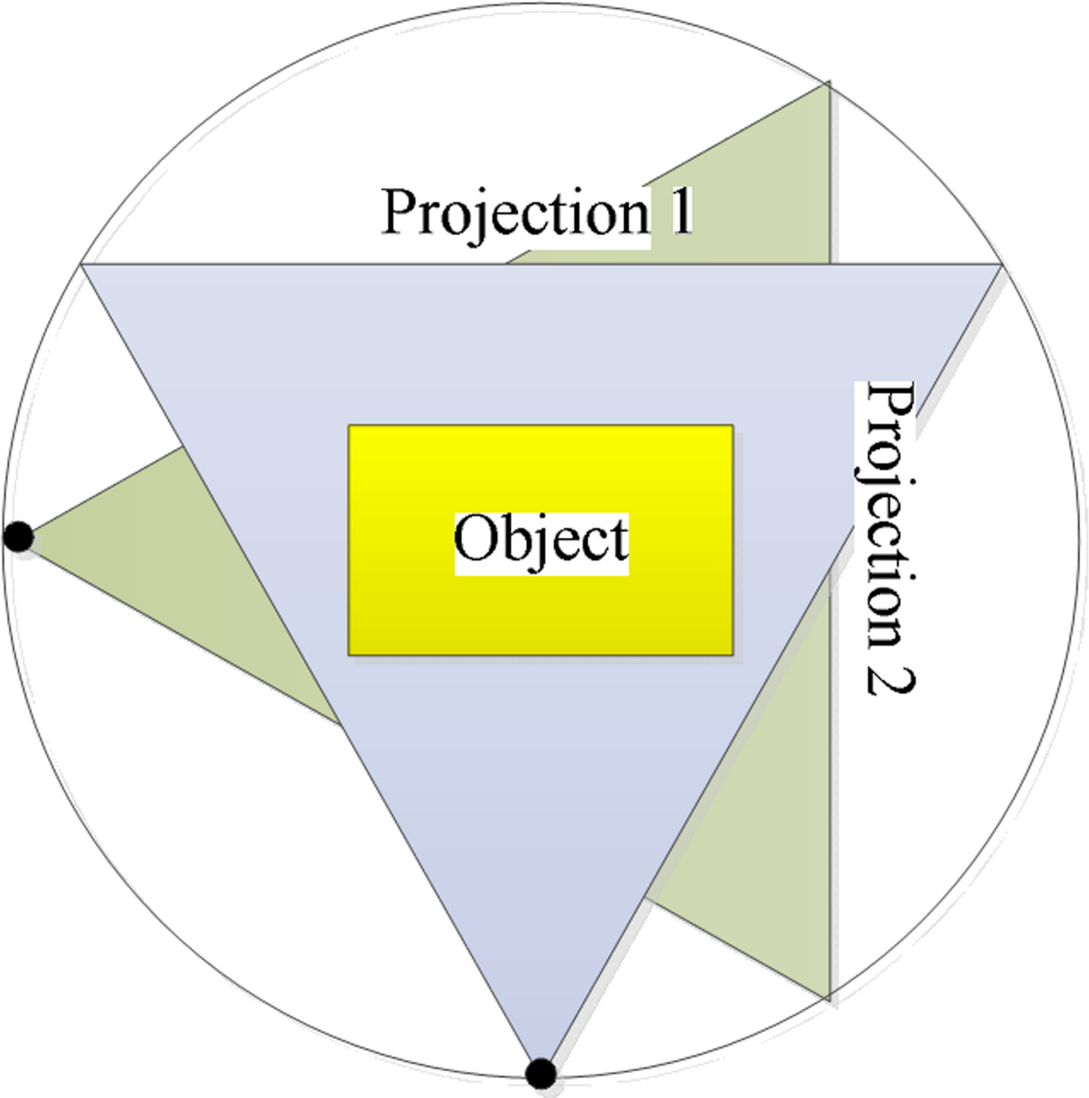
Circle trajectory scanning for a rectangular object.

Fig 1 illustrates a rectangular object for which the ray attenuation thickness of projection 2 is greater than that of projection 1. Increasing the length to width ratio increases the difference in the attenuation thickness. Therefore, using the traditional circle trajectory for a fixed X-ray energy results in the loss of projection information because one energy cannot balance two thicknesses with the limitation of the dynamic range about imaging detector. This problem can be solved using variable tube voltage imaging, which involves multi-imaging at different voltages and multi-spectrum fusion [3][4]. However, the radiation dose increases as a result. Therefore, we investigate changing the scanning mode. In the 3D-space of an irregular object, the thickness in some projection directions will match one value of the energy over a certain dynamic range. If these projection data have integrity, CT reconstruction will be realised. So we can define the 3D scanning space, relative to the traditional 2D scanning (such as circle trajectory, line trajectory) [5]. Here in order to guarantee the projection integrity and easy to engineering application, a spherical trajectory is called.

Recently, the development of C-arm CT has made the scanning trajectory highly flexible and practical, and significant progress has been achieved for image reconstruction from cone beam projections. Accurate and efficient reconstruction algorithms have been developed for various trajectories, e.g., helical [6], circle-and-line [7], and saddle [8]. Most of these algorithms have a filtered back-projection (FBP) structure. First, the cone beam projections are filtered, followed by back-projecting the result into the image space. Another, somewhat slower FBP algorithm that applies to a wide class of source trajectories can be found in Ye and Wang [9].

An interesting back-projection filtration (BPF) algorithm has been developed for helical CT [10]. This method can be generalised to other complete source trajectories [11]. Packet et al. [12] developed an excellent generalisation of this concept. However, the BPF structure is not very efficient from a computational perspective. One of the reasons for this inefficiency is that the back-projection step is time consuming. However, the BPF approach is much more flexible in handling truncated data than the FBP approach.

In particular, Gel’fand–Graev’s inversion formula reveals a fundamental relationship for the Hilbert transform along a chord in the field of view in terms of projection data [13]. This formula has wide applications in the CT field, including reconstruction from truncated projections and limited-angle tomography. Also the other scanning geometry, called computed laminography (CL), has been developed specifically for the imaging of flat samples [14][15]. In CL the rotation axis is tilted with respect to the incoming beam, the sample surface normal being approximately parallel to the rotation axis. In CL each projection has similar overall transmission, and all the projections can be recorded [16].

These new trajectories are used as references in this article but are not universal. Because of the complex of object structure, the scanning trajectory is not continuous, and it cannot be expressed with a single formula. So the above reconstruction algorithm is not applicative. To handle complex industrial structures, we develop a projection model and derive the projection matrix for scanning along a spherical surface based on knowledge of the space geometry. We also use an iterative reconstruction algorithm to prevent incomplete data from affecting the analytical algorithms.

## Trajectory planning

Consider an X-ray focal spot that is in 2D (non-linear, non-circular, or other type) motion on a plane or, more generally, in 3D motion within a neighbourhood and is facing a short object to be reconstructed; when the X-ray source is simultaneously rotated in a transverse plane of the object, the synthesised 3D scanning trajectory can take various forms with respect to the short object.

Specifically, let *r*>0 be the radius of the tube scanning circle on the spherical surface, where *θ* ∈ (0, *π*) and *s* ∈ (0, 2*π*) denote the angles of the two semi-axes of the scanning range in the plane of the focal spot that is facing the short object. Thus, we define a family of composite trajectories in the spherical surface as

$$\rho \left(r,\theta ,s\right)=\{\begin{array}{l} x=r\;\text{sin}\;\theta \;\text{cos}\;s\\ y=r\;\text{sin}\;\theta \;\text{sin}\;s\\ z=r\;\text{cos}\;\theta \end{array}.$$ (1)

Without loss of generality, we can rewrite (1) as

$$\rho \left(r,\theta ,s\right)=\{\begin{array}{l} x=r\;\text{sin}\;\theta \;\text{cos}\;s\\ y=r\;\text{sin}\;\theta \;\text{sin}\;s\\ z=r\;\text{cos}\;\theta \;\text{cos}\;\left(\omega s\right) \end{array},$$ (2)

where *s* ∈ *R* represents time. Several cases for this formula can be considered:

1. *θ* = *π* / 2 and *ω* = 0: the trajectory is a circle curve;
2. *θ* lies within (0, *π*) and *ω* ≥ 2: the trajectory is a saddle curve;
3. *θ* lies within (0, *π*) and *ω* = 1: the trajectory is an inclined circle curve;
4. *s* lies within (0, 2*π*) and *θ* ∈ (0, *π*): the trajectory is a half-baked inclined circle curve.

**Fig 2:**
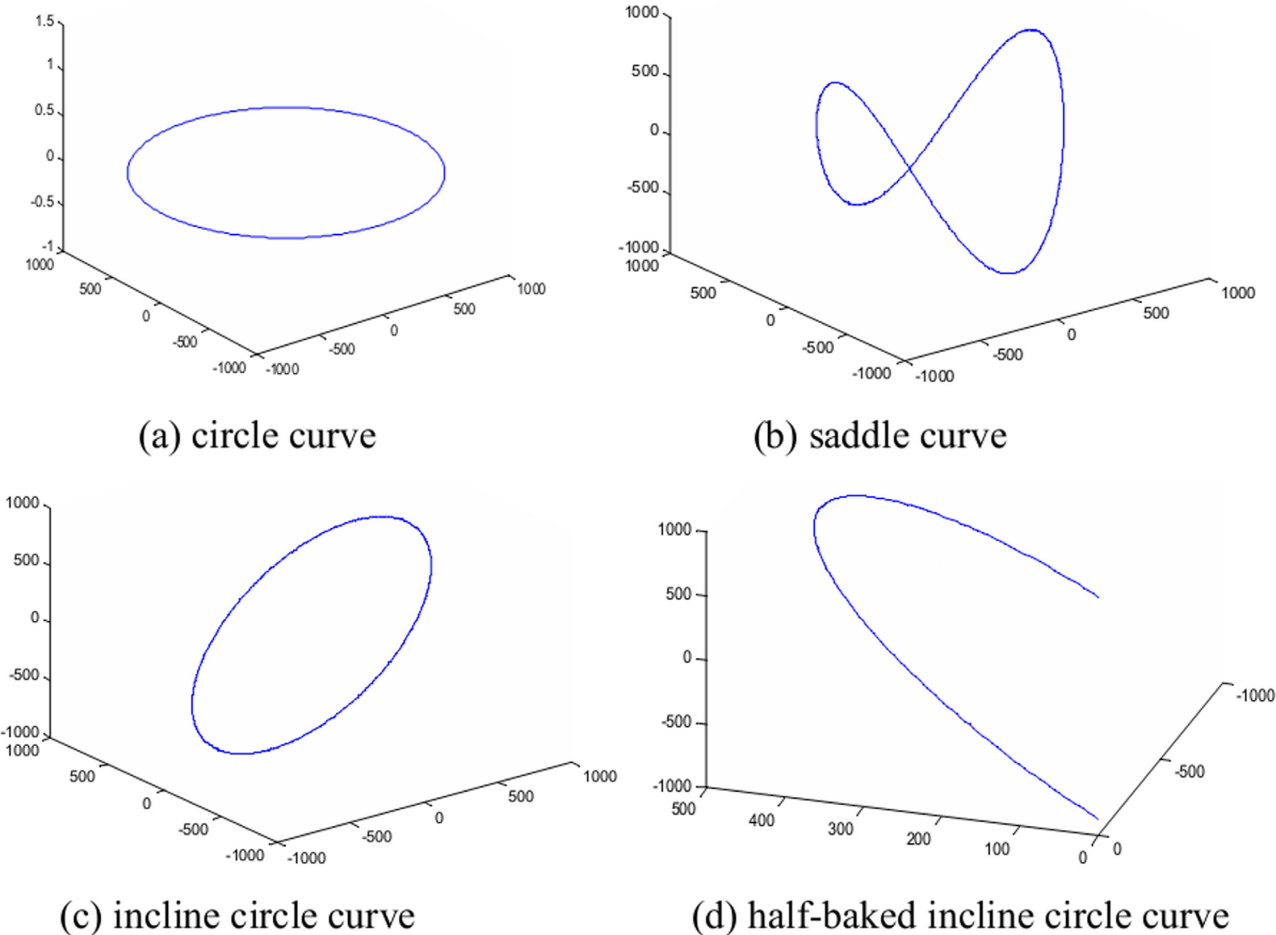
Scanning curves for different parameters on a spherical surface.

Some representative composite spherical surface curves are presented in Fig 2.

For the above the scanning curve, the traditional analytic reconstruction algorithm have no universality, which is main circle trajectory. However, the iterative reconstruction is the discrete. From the parallelism between ray path and imaging point in the detector, we can build the linear equation model. Then use iterative optimization to solve this equation. So in the above scanning trajectory, we can sample the discrete point at the scanning curve, and get the projection value based on projection geometry relationship. But we should get the projection matrix first.

## Projection matrix

In the iterative reconstruction algorithm, the imaging process is a discrete problem for the solution of linear equations:

AX=P, (3)

Where, *X* and *P* represent the column vector about the object’s linear attenuation coefficient and the projection data, respectively. *A* is the coefficient matrix that is determined by the relationship among the X-ray source, testing object and the detector. Each element of the coefficient matrix usually corresponds to a distance, i.e., the length of the X-ray through each pixel.

**Fig 3:**
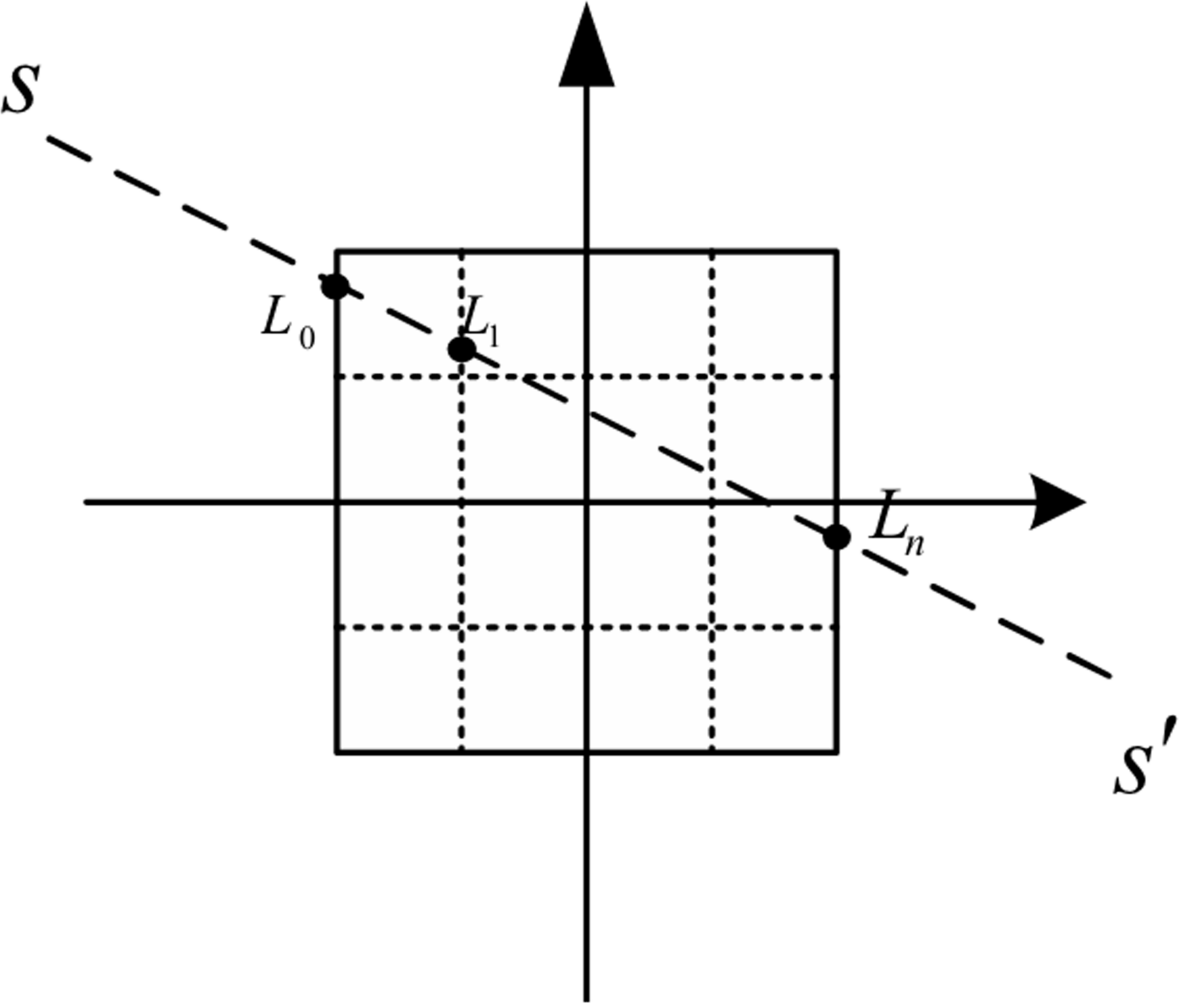
Geometrical relationship between a ray and an object.

Therefore, the projection matrix of a random trajectory can be obtained if the parameters about the projection direction and the positions of the X-ray source and the detector are confirmed. Fig 3 shows the X-ray source *ρ*(*s*), the point intersected by the X-ray and pixels *L*_*k*_, the first point where the ray enters the reconstruction region *L*_*0*_ and the first point *L*_*n*_ at which the ray leaves the reconstruction region.

We denote the position of the X-ray source in the image coordinate system by *s*(*x*_0_, *y*_0_, *z*_0_) and the projection point in the detector by $s^{\prime }\left(x_{0}^{\prime },y_{0}^{\prime },z_{0}^{\prime }\right)$. Then, we use the following linear formula

$$\frac{x-x_{0}}{x_{0}^{\prime }-x_{0}}=\frac{y-y_{0}}{y_{0}^{\prime }-y_{0}}=\frac{z-z_{0}}{z_{0}^{\prime }-z_{0}}=t$$ (4)

to determine the ray equation with the scanning curve (*R* cos(*s*), *R* sin(*s*), *R* cos*θ*cos(*ωs*)):

$$\frac{x-R\;\text{cos}\;\left(s\right)}{-d_{1}\;\text{sin}\;\left(s\right)-D\;\text{cos}\;\left(s\right)}=\frac{y-R\;\text{sin}\;\left(s\right)}{d_{1}\;\text{cos}\;\left(s\right)-D\;\text{sin}\;\left(s\right)}=\frac{z-R\;\text{cos}\;\theta \;\text{cos}\;\left(\omega s\right)}{d_{2}-2R\;\text{cos}\;\theta \;\text{cos}\;\left(\omega s\right)}=t,$$ (5)

where *D* is the distance from the source to the detector plane, and (*d*_1,_ *d*_2_) is the position of the detector pixel, which corresponds to a specified distance along the *xoy*-plane and the *z*-axis. Eq 5 is used to calculate the distance of the interaction between the ray and each small cube pixel with the traversal object’s pixel along the *x*-, *y*- and *z*-axes. These distances along the cube pixels correspond to the elements of the projection matrix.

## ART reconstruction algorithm

After the projection matrix is obtained, the ART algorithm can be used to reconstruct a CT image using a random trajectory for only complete projection data. The ART algorithm is

$$x_{j}^{\left(k+1\right)}=x_{j}^{\left(k\right)}+\lambda \frac{p_{i}-\sum_{n=1}^{N}a_{in}x_{n}^{\left(k\right)}}{\sum_{n=1}^{N}a_{in}^{2}}a_{ij}\;,$$ (6)

where *k* is the number of iterations, *a*_*ij*_ is the coefficient of the projection matrix, *p*_*i*_ is the vector of the projection data, *x*_*j*_ is the value of the *j*-*th* pixel in the reconstructed image, and *λ* ∈ (0, 2) is the relaxation factor.

Eq 6 shows that the scanning changes only affect the size of the weighting factor and do not affect the iterative algorithm. Therefore, we describe the procedure used to execute the iterative algorithm and extend the ART to solve the spherical trajectory CT reconstruction.

## Simulation experiment and discussion

The spherical trajectory corresponds to the general case. In this scanning mode, we obtain redundant information that adversely affects the reconstruction efficiency and quality. A projection point on the spherical trajectory should be selected based on the construction and shape of the object to prevent redundancy in the data.

Various problems are encountered using traditional circle trajectory scanning. For example, large cone angle scanning causes projection loss on both ends of the object’s *z*-axis, limited angle scanning causes the edge of the CT image to appear fuzzy, and a tabular object cannot be used to obtain projection information with integrity because of the mismatch between the ray energy and the thickness. These problems all result from using the circle trajectory, which causes projection loss. Therefore, we modify the circle trajectory. Here, we use an inclined circle trajectory as an example. This trajectory can be used to obtain more information at both ends of the *z*-axis for a large-angle cone; thus, more information can be obtained for a limited angle to match the ray energy to the thickness of a tabular object.

We also use the well-known 3D Shepp-Logan head phantom to demonstrate the effectiveness of the method and use a tabular object to demonstrate that the new scanning method can compensate for the projection loss. The equation of a circle is

$$\rho \left(R,s\right)=\{\begin{array}{l} x=R\;\text{cos}\left(\text{s}\right)\\ y=R\;\text{sin}\;\left(s\right)\\ z=0 \end{array}.$$ (7)

The equation of an inclined circle is

$$\rho \left(R,s,h\right)=\{\begin{array}{l} x=R\text{cos}\left(\text{s}\right)\\ y=R\;\text{sin}\;\left(s\right)\\ z=h\;\text{cos}\;\left(s\right) \end{array}.$$ (8)

Here, *R* is the distance between the X-ray source and rotation centre, *D* is the distance between the rotation centre and the detector, and *h* is the maximum height of the inclined circle along the *z*-axis. These two trajectories are used to analyse the reconstruction results for different scenarios.

### Small cone angle scanning

Here, *R* = 1000, *D* = 2000 and *h* = 400. The units are in pixels. The image size of the model and the reconstruction CT are 128×128. The same parameters are used to obtain the images shown below.

**Fig 4:**
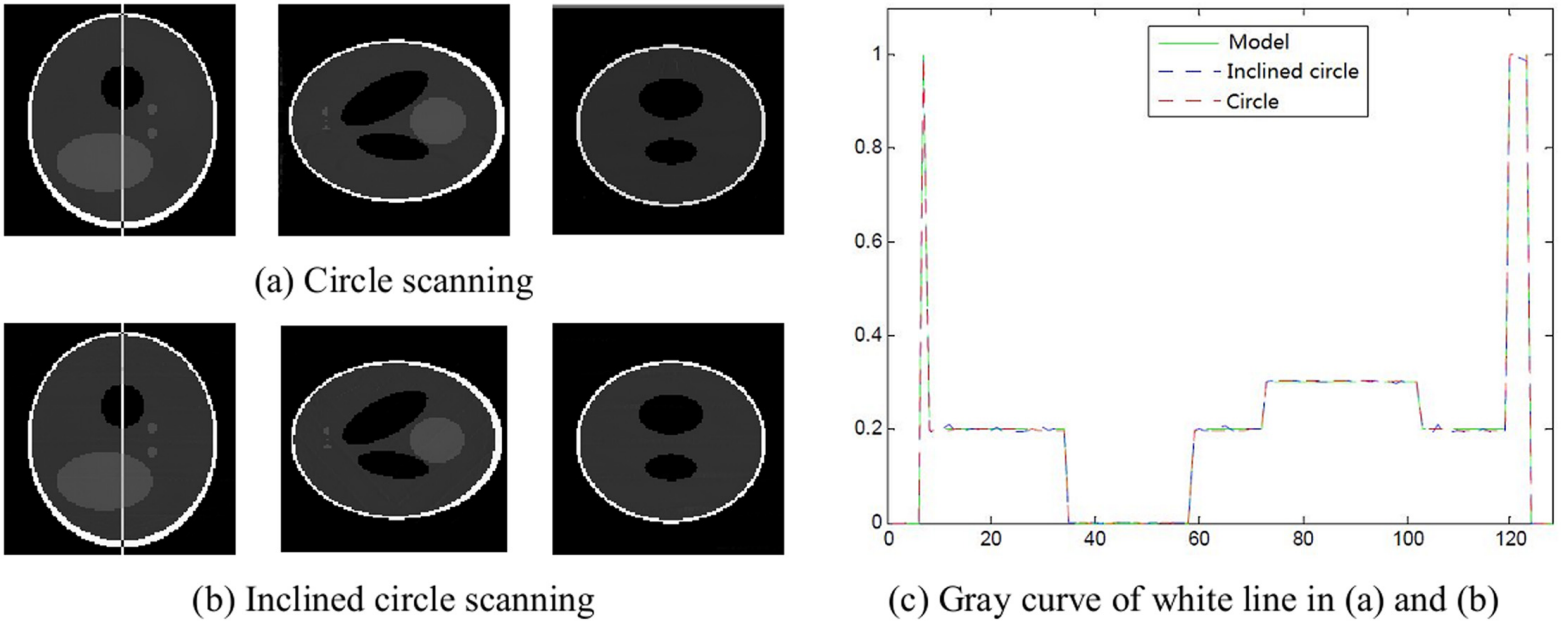
Slices of reconstruction image with small cone angle.

Fig 4 shows that the circle scanning and the inclined circle scanning can both reconstruct the object well. In addition, compared with the gray curve, these two scanning modes both approach the model image.

### Larger cone angle scanning

**Fig 5:**
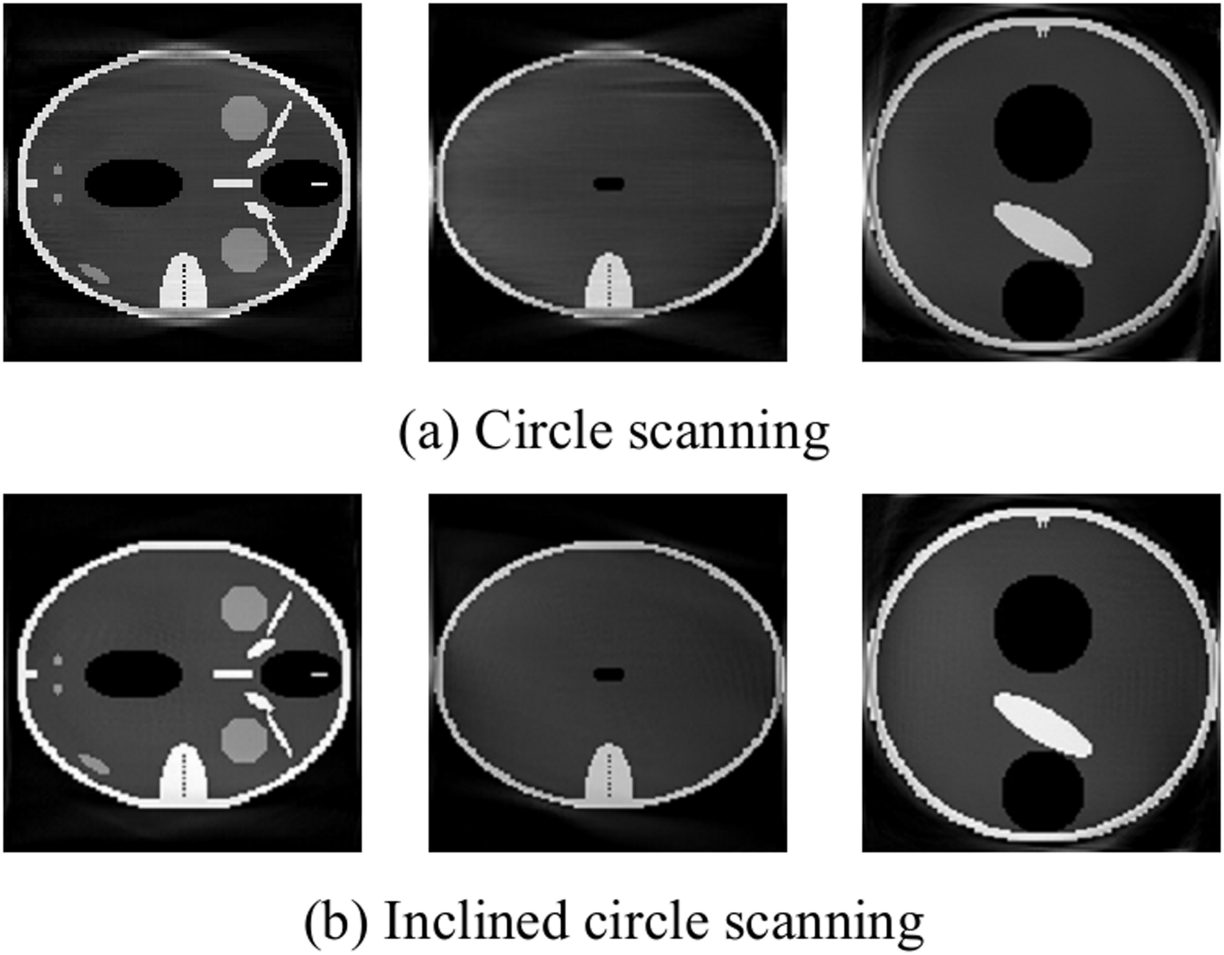
Slices of reconstruction image with larger cone angle.

Here, scanning is performed at larger cone angles, such as 36°, with *R* = 192, *D* = 384 and *h* = 64. The image is then reconstructed using circle and inclined circle scanning: the central slice is shown in Fig 5.

Fig 5(A) and 5(B) show that the two trajectories can both reconstruct the image; however, data are randomly missed as the cone angle increases: the instances of missing data are more significant for the larger cone angle [17]. For example, when the cone angle is *π*/5, the projection data are less complete, and the reconstruction image has fuzzy edge information. However, using inclined circle scanning, the missing data can be compensated for by adjusting the value of the *z*-direction, and the reconstruction image has sharpened edges and a high contrast. Namely, the inclined circle scanning produces superior results for a large cone angle than the circle scanning. Therefore, we can modify the scanning trajectory to compensate for the missing projection data to enhance the image reconstruction quality.

### Limited angle reconstruction

**Fig 6:**
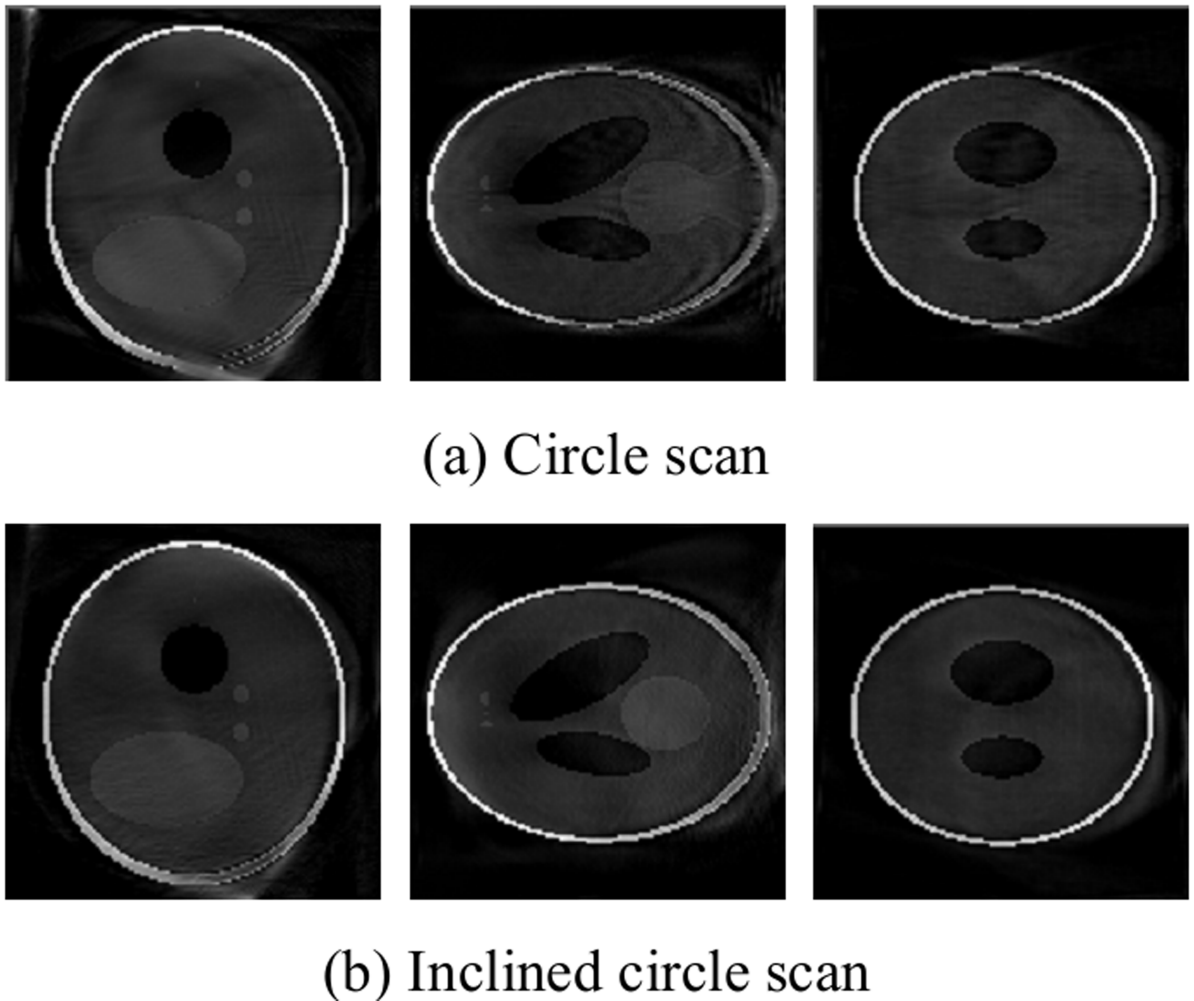
Slices of reconstruction image with limited angle.

Here, scanning parameters with *R* = 192, *D* = 384 and *h* = 64 is used, and the sample projection angle is only 140 degree. The reconstruction results, which are obtained by circle and incline circle scanning is shown in Fig 6.

Fig 6 shows that for incomplete projection, the reconstruction image obtained using inclined circle scanning is superior to that obtained using circle scanning, because some of the data of the projection angle are compensated for by changing the value of the z-direction and improving the reconstruction of both ends of the projection data obtained by the circle scanning.

### Tabular object reconstruction

**Fig 7:**
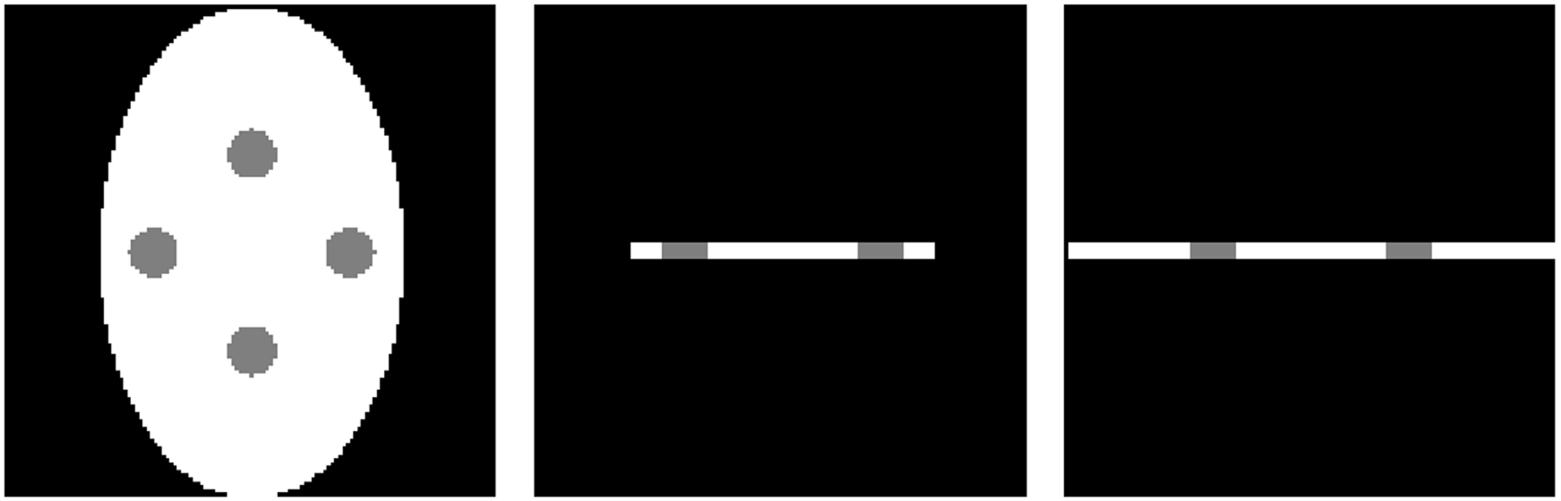
Tabular object phantom slices.

**Fig 8:**
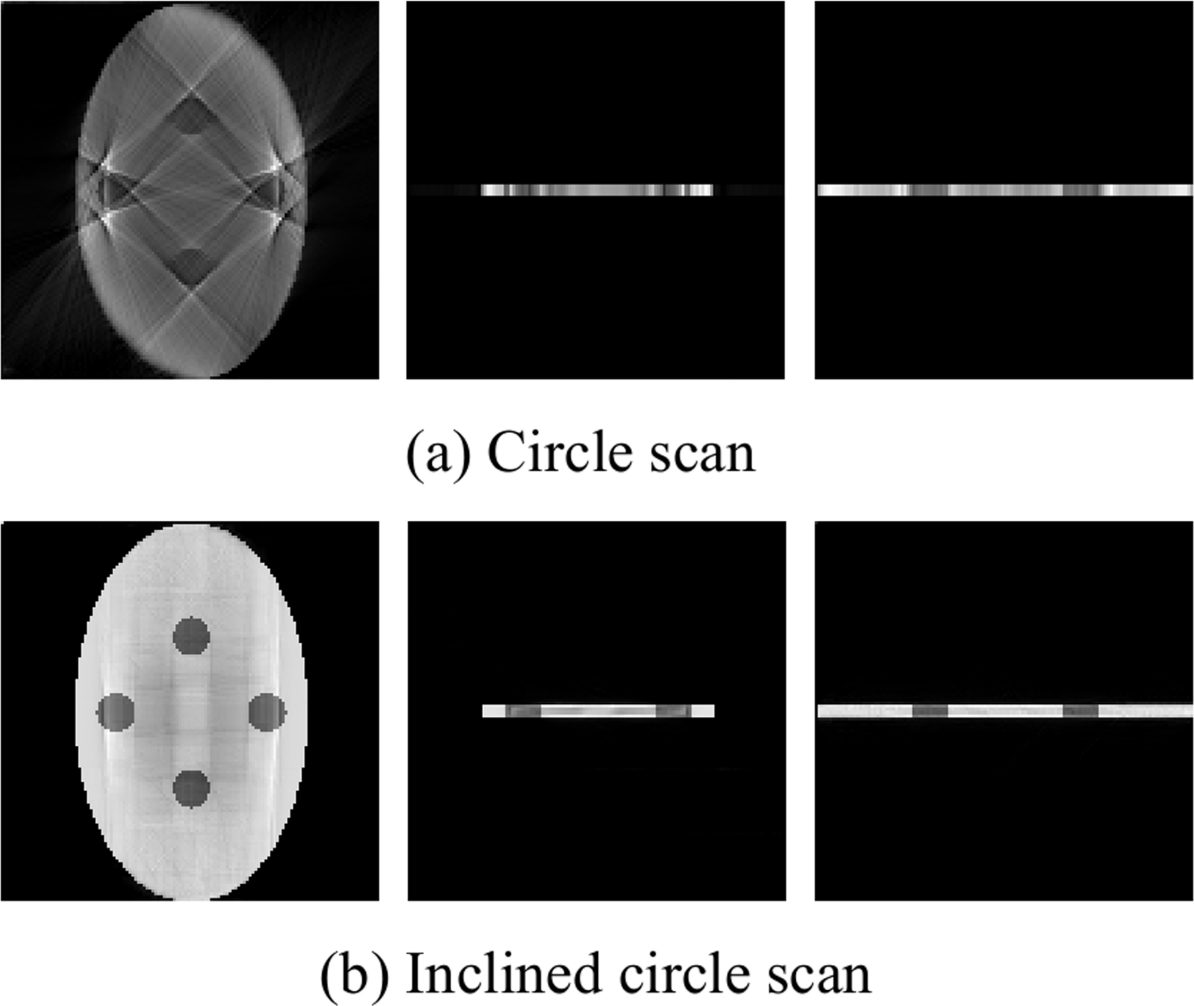
Slices of reconstructed image with tabular object.

In our simulation, the tabular object phantom size is 128×80×4, and the slices are shown in Fig 7. Using small cone-beam scanning with *R* = 1000, *D* = 2000 and *h* = 400, the reconstructed image is formed using circle and inclined circle scanning. The projection and central slice are shown in Fig 8.

The thickness of the tabular object is usually thinner in the *z*-direction than in the other directions, and the length to width ratio is usually larger in the *xoy*-plane than the other planes. The traditional fixed energy scanning model produces a mismatch between the ray energy and the transmission at every projection angle, which results in vague reconstruction information, as shown in Fig 8(A).

However, adjusting the value of the *z*-direction can compensate for the missing projection data with the inclined circle scanning model (Fig 2(C)). Thus, a smaller and more consistent transmission thickness is obtained that can match the energy. Then, we can obtain more complete projection data and reconstruct a high-contrast CT image, as shown in Fig 8(B). Therefore, a high-quality CT image of a tabular object can be effectively reconstructed by changing the scanning trajectory to compensate for missing projection data. This method can also be used to solve the CT imaging of a circuit board.

## Conclusions

In this paper, we develop spherical trajectory scanning to solve the problems of projection loss that arise from irregular object CT imaging, because of a mismatch between the ray energy and the thickness using the traditional circle trajectory. For the complex object, considering the universality of the spherical trajectory and data redundancy, the some discrete scanning points only can be selected. Then derive a projection matrix model for a spherical trajectory and use ART to reconstruct image along the discrete scanning points. We perform a simulation to address some of the problems that are encountered using the traditional circle trajectory, such as a large cone angle, a limited angle, and a tabular object. The experiments show that our method can ensure data integrity for projections and produces superior results for large cone angles, limited angles and tabular objects than the traditional method.

In order to further improve the reconstruction quality, we can add some constraint conditions to ART algorithm, such as total variation (TV), penalty function, and so on. Also, we can research the new reconstruction algorithm based on this new scanning mode.
